# Association of early life adversity with internalizing and externalizing behavior across 3 low- and middle-income countries

**DOI:** 10.1186/s13034-026-01139-9

**Published:** 2026-07-24

**Authors:** Sheri-Michelle Koopowitz, Richard Shadwell, Nadia Hoffman, Heather J. Zar, Giovanni Salum, Pedro M. Pan, Gauri Divan, Supriya Bhavnani, Dan J. Stein

**Affiliations:** 1 Neuroscience Institute, Faculty of Health Sciences, University of Cape Town, Cape Town, South Africa; 2 South African Medical Research Council (SAMRC) Unit on Risk and Resilience in Mental Disorders, Cape Town, South Africa; 3 Department of Paediatrics and Child Health, Red Cross War Memorial Children’s Hospital, University of Cape Town, Cape Town, South Africa; 4 South African Medical Research Council (SAMRC) Unit on Child and Adolescent Health, Cape Town, South Africa; 5 Global Programs, Child Mind Institute, New York, USA; 6 Universidade Federal do Rio Grande do Sul, Hospital de Clínicas, Porto Alegre, Brazil; 7 Universidade Federal de São Paulo (UNIFESP), São Paulo, Brazil; 8 Child Development Group, Sangath, India

**Keywords:** Behavior, Cohort studies, Childhood, Brazil, India, South Africa

## Abstract

**Background:**

Early life adversity (ELA) is associated with increased internalizing and externalizing behavior in high-income cohorts. However, it is unclear whether these associations generalize to low- and middle-income countries (LMICs). We examined the association between ELA and internalizing and externalizing behaviors in children across three LMIC cohorts.

**Methods:**

Data from three cohorts, the Brazilian High-Risk Cohort (BHRC; *n* = 1111), Sustainable Programme Incorporating Nutrition and Games (SPRING; *n* = 601), and Drakenstein Child Health Study (DCHS; *n* = 708) were pooled. Children (7–10 years) were assessed using the Strengths and Difficulties Questionnaire (SDQ). ELA was measured using a cumulative index. Ordinary Least Squares linear regression models were run to examine the associations of adversity with internalizing and externalizing behaviors while adjusting for child age, sex, and cohort, and to test interactions with child age, sex, and cohort. 2420 children (53.9% boys) were included in analyses.

**Results:**

SDQ measurement invariance was not established across cohorts. ELA associations were strongest in BHRC (SDQ total: B = 6.78, *p* < .001; internalizing: B = 3.3, *p* < .001; externalizing: B = 3.48, *p* < .001), attenuated in SPRING (SDQ total: B = 1.54, *p* = .014; internalizing: B = 0.81, *p* < .001; externalizing: B = 0.73, *p* = .007), and not observed in DCHS (SDQ total: B = 0.12, *p* = .78; internalizing: B = -0.18, *p* = .42; externalizing: B = 0.3, *p* = .29). Interaction analyses indicated cohort heterogeneity and age-related effects, with inconsistent sex moderation across cohorts and outcomes.

**Conclusions:**

In Global South cohorts, while there is an association between ELA and psychopathology, there are also important context-dependent differences across cohorts.

**Supplementary Information:**

The online version contains supplementary material available at https://doi.org/10.1186/s13034-026-01139-9.

## Background

Early life adversity (ELA) is a determinant of child neurodevelopment, mental health, and behavior with effects that persist across the lifespan [1–4]. ELA encompasses childhood adversities (e.g., abuse, neglect), socioeconomic adversities (e.g., poverty, incomplete education), maternal stress exposures (e.g., traumatic events), and relationship adversities (e.g., family conflict) [5]. Such experiences may disrupt brain maturation during sensitive developmental periods, shaping trajectories of internalizing and externalizing behavior [1, 2, 6]. Evidence suggests that exposure to multiple, cumulative adversities in the first years of life may have dose-dependent effects [7, 8]. This is particularly important in low- and middle-income countries where children experience a variety of adversities in their early years [5, 9]. Approximately one-third of adult mental disorders are attributable to childhood adversity [3], and in children, ELA is associated with internalizing or externalizing behaviors that often precede later psychiatric disorders [2, 10]. Behavior varies systematically across development, with externalizing difficulties tending to be more prominent in early childhood and internalizing symptoms increasing in relative prominence during later childhood and adolescence [11, 12]. Sex differences are also observed, with boys exhibiting greater externalizing behavior and girls exhibit greater internalizing behavior [13].

The majority of research on associations of early life adversity and child behavior has been conducted in high-income countries [14], despite most of the world’s children living in low- and middle-income countries (LMICs) [10]. In low- and middle-income countries, children are more frequently exposed to structurally embedded adversities such as community violence, poverty, and food insecurity [15, 16], which may differ from adversities more commonly studied in high-income settings, such as maltreatment and household dysfunction [17]. Although evidence is limited, studies from LMIC cohorts consistently show that higher adversity exposure is associated with greater emotional and behavioral problems [18, 19]. In LMICs, children exposed to adversity exhibit more behavioral problems at a younger age [19]. In particular, LMIC children exposed to cumulative adversity exhibit emotional and conduct problems at age 7 [18]. Across settings, effect sizes appear modest but directionally consistent. The extent to which the findings from high-income settings can be generalized elsewhere is unclear.

Evidence further indicates heterogeneity in associations between early life adversity and later behavioral and mental health outcomes, with significant moderation by socioeconomic context and country income level [20–22]. Although Brazil, India, and South Africa differ in socioeconomic conditions, cultural context, and adversity profiles, developmental theory supports partial cross-context convergence in mechanisms linking early adversity to child behavior. Dimensional models of adversity suggest that heterogeneous exposures map onto broader constructs with partially conserved neurodevelopmental effects across contexts [23]. Therefore, pooling data across these settings can be interpreted as estimating associations that reflect shared underlying mechanisms operating within distinct contextual environments [24]. Furthermore, the focus on adversity in the first two years reflects a sensitive period, during which rapid brain development may confer heightened vulnerability to environmental inputs and experiences [25, 26]. This study therefore examines whether cumulative early life adversity in the first two years of life is associated with internalizing and externalizing behaviors in children across three LMIC cohorts.

## Methods

### Aims

The aim of this study was to examine the associations of early life adversity with child internalizing and externalizing behavior at 7–10 years using pooled behavioral data from three low- and middle-income cohorts. We hypothesized that higher exposure to early life adversity would be associated with greater internalizing and externalizing difficulties across cohorts. We further hypothesized sex differences in behavioral outcomes, with differential patterns between boys and girls, and an age effect such that younger children would show greater behavioral difficulties.

### Sample and cohorts

Data were collected from children enrolled in three low- and middle-income country cohorts. As each cohort conducted assessments at different time points, outcome data from children aged 7–10 years were utilized in this study.

The Brazilian High-Risk Cohort (BHRC) is a large community school-based study that has followed 2511 children from Brazil since 2010. Psychological, genetic, and neuroimaging data were collected with the aim to investigate typical and atypical trajectories of psychopathology and cognition over development. A two-stage design was used. First, childhood symptoms and family history of psychiatric disorders were assessed in a screening interview, collecting information from 9937 index children at 57 schools in the cities of São Paulo and Porto Alegre, as well as from 45 394 family members. In the second stage, a random subsample (intended to be representative of the community, *n* = 958) and a high-risk subsample (children at increased risk for mental disorders, based on family risk and childhood symptoms, *n* = 1554) were selected for further evaluation. These 2511 subjects were evaluated using an extensive protocol, involving one 2-hour home evaluation with the parents, two 1-hour evaluations of the child by a psychologist, and two 1-hour evaluations of the child by a speech pathologist [27]. The sample is non-probabilistic and drawn from urban state-funded schools, limiting generalizability, and the cohort’s focus on mental health further constrains generalizability [28]. Data from *n* = 1111 BHRC participants were included in this study. Behavioral outcomes were restricted to ages 7–10 to align developmental stages across cohorts and maximize comparability of SDQ assessments.

The SPRING (Sustainable Programme Incorporating Nutrition and Games) study is a cluster randomized controlled trial that was conducted in the Rewari district of Haryana, India– a predominantly rural region with the largest number of young children at extreme risk of impaired cognitive and social-emotional development [29, 30]. 7015 families were enrolled by the surveillance system from 24 clusters, defined as the catchment area of a functional primary health sub-center. Trial outcome measures were assessed in 1443 children at 18 months of age [31]. SPRING-ELS (Early Life Stress) was a nested sub-study of the SPRING trial evaluating the effects of early adversities and stress on child growth and development [5]. Most recently, through the COINCIDE study, children were followed up at 8 years of age and data from *n* = 601 participants were included in this study. The study setting is diverse, but extended joint-family households are common, with multiple adult caregivers (such as parents, aunts/uncles, grandparents) involved in child-rearing [30].

The Drakenstein Child Health Study (DCHS) is a multidisciplinary birth cohort study that drew its participants from two peri-urban, relatively stable, low socioeconomic communities in the Western Cape, South Africa [32]. Within this region, individuals live in informal housing or crowded conditions, and experience high levels of unemployment [32]. Pregnant women between 20- and 28-weeks’ gestation were recruited from two primary health care clinics for the main DCHS study. Women were eligible for the study if they were 18 years or older, planned attendance at one of the two recruitment clinics, and intended to remain in the area. During a three-year recruitment period (March 2012 to March 2015), 1225 pregnant women were enrolled into the DCHS antenatally; 88 (7.2%) mothers were lost to follow up antenatally, had a miscarriage or a stillbirth. In total, 1137 women gave birth to 1143 live infants (4 twins and 1 triplet). Data from *n* = 708 DCHS participants were included in this study.

### Materials

The Strengths and Difficulties Questionnaire (SDQ) is a brief behavioral screening questionnaire that can be used for children between 2 and 17 years old. The SDQ measures 25 attributes designed to measure emotional problems (5 items), conduct problems (5 items), hyperactivity/inattention (5 items), peer problems (5 items), and prosocial behaviors (5 items) using a 3-point Likert scale [33, 34]. Responses for each question range from “not true” being scored 0 points, “somewhat true” being scored 1 point, and “certainly true” being scored 2 points. Total scores can be generated for the individual subscale or an overall total score. To generate a total difficulties score, emotional problems, conduct problems, hyperactivity/inattention, and peer problems are added together [34]. To measure internalizing problems, the emotional problems and peer problems subscales are summed together, while externalizing problems are measured using the conduct problems and hyperactivity/inattention symptoms subscales [35]. The SDQ Parent Report Measure for Children was utilized by all three cohorts, and each cohort utilized the versions suitable for the cohort (BHRC: Brazilian Portuguese; SPRING: Hindi; DCHS: Afrikaans and isiXhosa). The SDQ has acceptable reliability and validity across Western and non-Western settings [36], and has been used in research settings in Brazil [37], India [38], and South Africa [39].

To facilitate comparisons across cohorts, adversity scores were standardized within each sample. Using the SPRING cohort adversity variable [5] as a template, the individual cohorts created an adversity variable using equivalent measures that reflected the nuances of the individual cohorts. The SPRING adversity variable was chosen due to the focus on early life adversity – adversity taking place within the first year of the child’s life and has been used in relation to growth and development outcomes in the SPRING cohort [5]. This variable measured 22 adversities across 4 groups of adversities: socioeconomic adversities, maternal stress adversities, relationship adversities, and child adversities [5]. Socioeconomic adversities include debt, parental education, and parental occupation. Maternal stress adversities include psychosocial risk factors such as maternal depression symptoms, exposure to IPV, and family support. Relationship adversities include difficulties in caregiver-child relationships and parenting and bonding risk factors. Child adversities include child risk factors such as prematurity, NICU hospitalizations, and mother and child separated in the first year of life [5]. Variable selection was conducted at the domain level by mapping conceptually equivalent variables within each cohort, prioritizing conceptual over item-level equivalence. For example, where the Duke Social Support and Stress Scale was unavailable, cohort-specific measures capturing comparable domains of perceived support and interpersonal stress were selected.

**Fig. 1:**
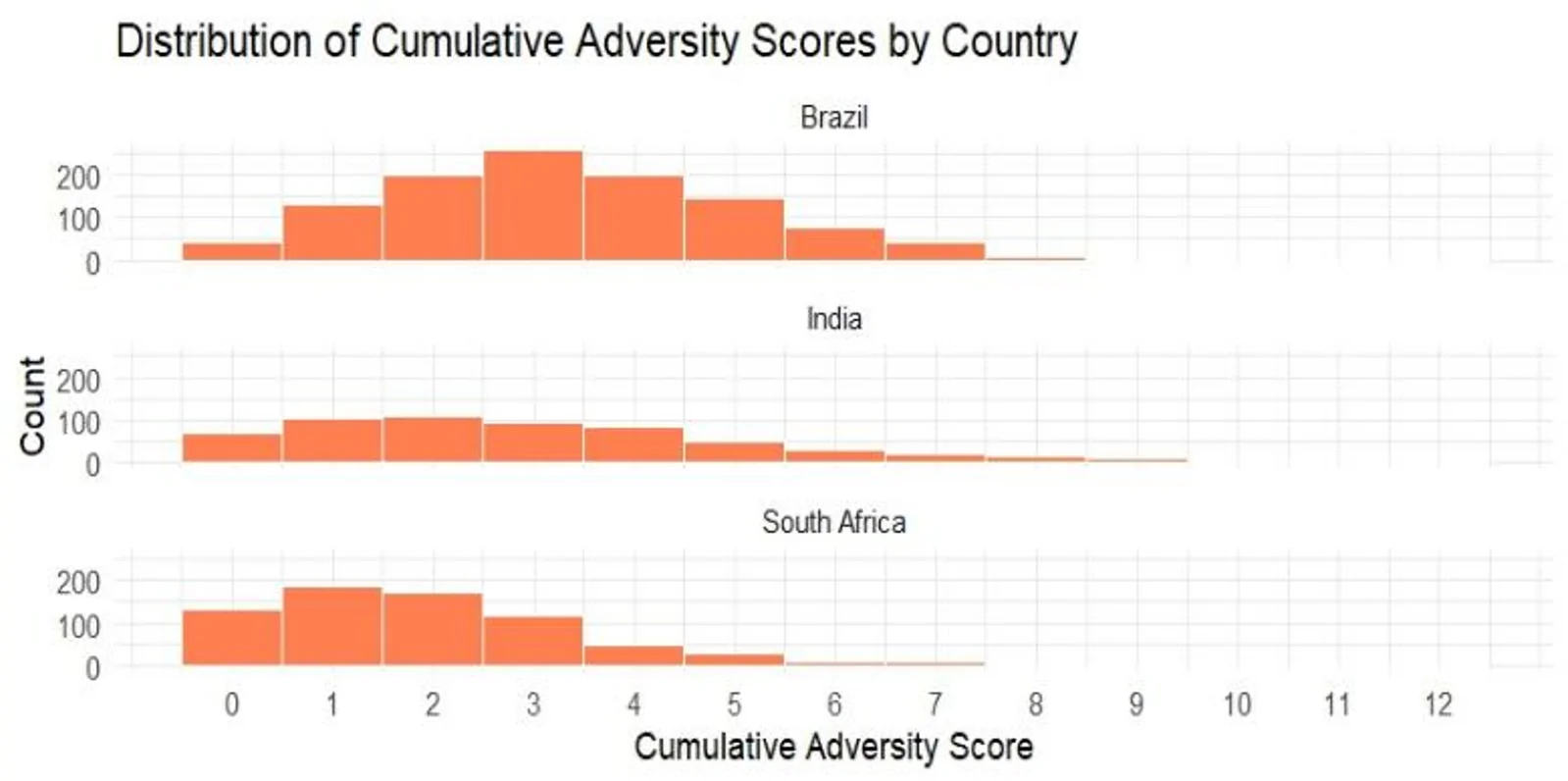
Distribution of cumulative adversity scores by country

Adversity scores ranged from 0 to 22 in the SPRING cohort and from 0 to 12 in the BHRC and DCHS cohorts. Adversity variables per cohort can be found in supplementary materials, and Fig. 1 demonstrates the adversity distribution per cohort. Where exact item matches were not possible, cohort-specific variables were selected to reflect the same adversity domains; any exposure was then dichotomized (0 = no ELA exposure, 1 = any ELA exposure) for the primary analyses. There were no missing data for adversity variables in the SPRING cohort. In contrast, some adversity items were missing in the BHRC and DCHS cohorts; these were coded as absence of exposure (0) rather than imputed, under the assumption that non-response reflected non-endorsement of the adversity item. Complete-case exclusion would have reduced the sample and risked introducing additional bias in the adversity variable. The early life adversity variable reflects cumulative exposure up to age 2 and does not capture the persistence of adversities beyond this period.

### Data analysis

Data were analyzed using *R* [40]. The association between early life adversity and internalizing and externalizing behaviors was examined using multiple linear regression models, controlling for child age, child sex, and cohort, using complete-case analyses. SDQ measurement invariance (MI) was used to test where the optimal measurement model was equivalent across the cohorts. Measurement invariance indicates the extent to which a measure is interpreted in the same way across different groups of individuals, and without measurement invariance, the measure may be subject to measurement errors [41]. To test measurement invariance, R’s *lavaan* was used. If measurement invariance did not hold, we planned to conduct within-cohort analyses using: (1) multiple linear regression models with child age and child sex as covariates, and (2) linear models with interaction terms included. We report unstandardized coefficients (B) in-text and standardized coefficients (β) in the tables.

## Results

### Measurement Invariance

SDQ measurement invariance was tested sequentially across cohorts. The configural model (no equality constraints) provided a borderline baseline fit (TLI = 0.888). Imposing metric invariance constraints significantly worsened the model fit compared to the configural model, χ²(32) = 729.8, *p* < .001, with TLI decreasing to 0.884. Further constraining the model to scalar invariance also resulted in a significant decrease in fit relative to the metric model, χ²(32) = 810.1, *p* < .001, with TLI decreasing to 0.854. As scalar invariance was not achieved, pooled effect estimates may be biased; therefore, we present within-cohort regression results in addition to pooled analyses.

### Sample characteristics

Sample characteristics can be found in Table 1. A total of *N* = 2420 participants were included in the analyses with 53.9% boys. The mean child’s age was 8.3 years (*SD* = 0.7). Missing age data was imputed with mean imputation.

The Brazilian High-Risk Cohort included a total of *n* = 1111 participants in this study. 1400 children (aged 11–14 years) did not meet the 7–10-year age range criteria and were excluded from analysis. No participants were excluded due to missing data. The mean child age was 8.6 years (*SD* = 0.8), and over half of this cohort (56.3%) were boys.

The Sustainable Programme Incorporating Nutrition and Games cohort included a total of *n* = 601 participants in this study. No participants were excluded due to missing data. The mean child age was 7.9 years (*SD* = 0.3), and over half of this cohort (54.6%) were boys.

The Drakenstein Child Health Study cohort included a total of *n* = 1143 participants in this study. After excluding *n* = 435 participants due to incomplete or missing data, analyses included *n* = 708 participants. The mean child age was 8.0 years (*SD* = 0.2), and under half of this cohort (49.7%) were boys. Participants included in the DCHS analytic sample differed from those excluded due to missing data on the following variables: included participants had higher maternal HIV prevalence, lower maternal education, higher asset scores, and fewer preterm births. Detailed comparisons are provided in the Supplementary Materials.

The cohorts differed significantly in child age (*F*(2, 2417) = 352.5, *p* < .001), with BHRC an older cohort than both SPRING (*p* < .001) and DCHS (*p* < .001). The cohorts differed in child sex (*χ*^*2*^ = 7.8, *p* = .02), with more boys in BHRC than SPRING and DCHS.

**Table 1:** Sample characteristics

	Full sample	BHRC	SPRING	DCHS		
	*N* = 2420	*n* = 1111	*n* = 601	*n* = 708	F or χ^2^	*p*
Age	8.3 (0.7)	8.6 (0.8)	7.9 (0.3)	8.03 (0.2)	352.5	< 0.001
Male %	53.8%	56.3%	54.6%	49.7%	7.8	0.02
SDQ total score	10.3 (7.5)	14.8 (7.8)	4.1 (3.5)	8.5 (4.5)	655.7	< 0.001
Internalizing	4.4 (3.9)	6.8 (4.1)	1.8 (1.9)	2.9 (2.3)	608.1	< 0.001
Externalizing	5.9 (4.5)	7.9 (4.9)	2.3 (2.2)	5.6 (2.9)	437.1	< 0.001

SDQ denotes Strengths and Difficulties Questionnaire. F tests for continuous variables and Chi Square for categorical variables

## Primary findings: cohort-specific findings

Due to measurement invariance not holding, within-cohort analyses were run. Pooled analyses were exploratory in nature and can be found in Supplementary Materials. Cohort-specific analyses revealed both consistent and divergent patterns in the relationship between early life adversity and internalizing and externalizing behaviors. See Table 2 for the standardized estimates (β) for each model.

**Table 2:** Cohort-specific findings

		SDQ total				Internalizing				Externalizing			
Country	Variable	Estimate β	CI Lower	CI Upper	p value	Estimate β	CI Lower	CI Upper	p value	Estimate β	CI Lower	CI Upper	p value
Brazil	Adversity	0.17	0.11	0.23	< 0.001 ***	0.16	0.10	0.22	< 0.001 ***	0.14	0.08	0.20	< 0.001 ***
	Age	0.01	– 0.05	0.07	0.81	0.03	– 0.03	0.09	0.28	– 0.02	– 0.07	0.04	0.60
	Sex (Girl)	– 0.15	– 0.26	– 0.03	0.014 **	0.00	– 0.12	0.12	0.99	– 0.24	– 0.35	– 0.12	< 0.001 ***
India	Adversity	0.14	0.07	0.22	< 0.001 ***	0.14	0.06	0.22	< 0.001 ***	0.11	0.03	0.18	0.007 **
	Age	– 0.31	– 0.39	– 0.24	< 0.001 ***	– 0.25	– 0.32	– 0.17	< 0.001 ***	– 0.28	– 0.36	– 0.21	< 0.001 ***
	Sex (Girl)	– 0.08	– 0.23	0.07	0.31	– 0.01	– 0.17	0.14	0.90	– 0.11	– 0.27	0.04	0.15
South Africa	Adversity	0.01	– 0.06	0.08	0.79	– 0.03	– 0.10	0.04	0.42	0.04	– 0.03	0.11	0.29
	Age	– 0.20	– 0.27	– 0.13	< 0.001 ***	– 0.11	– 0.19	– 0.04	0.003 **	– 0.21	– 0.28	– 0.14	< 0.001 ***
	Sex (Girl)	– 0.27	– 0.42	– 0.13	< 0.001 ***	– 0.15	– 0.30	– 0.01	0.04 *	– 0.30	– 0.44	– 0.15	< 0.001 ***

Coefficients are standardized regression estimates (β). SDQ denotes Strengths and Difficulties Questionnaire; CI denotes 95% confidence intervals

* denotes p < .05; ** denotes p < .01, *** denotes p < .001

In BHRC, early life adversity showed strong evidence of an association with higher SDQ scores (B = 6.78, 95% CI 4.49, 9.07, *p* < .001). Girls had lower SDQ scores than boys (B = – 1.14, 95% CI – 2.06, – 0.23, *p* = .014). In SPRING, adversity also predicted higher SDQ scores (B = 1.54, 95% CI 0.72, 2.35, *p* < .001). Each additional year of age was associated with lower SDQ scores (B = – 3.23, 95% CI – 4.01, – 2.45, *p* < .001). In DCHS, adversity was not associated with SDQ scores (B = 0.12, *p* = .78). However, each additional year of age was associated with lower SDQ scores (B = – 5.5, 95% CI – 7.50, – 3.49, *p* < .001), and girls scored lower than boys (B = – 1.23, 95% CI – 1.87, – 0.59, *p* < .001).

In BHRC, early life adversity was associated with higher internalizing scores (B = 3.3, 95% CI 2.10, 4.50, *p* < .001). Neither age nor sex were significant predictors. In SPRING, early life adversity was positively associated with internalizing scores (B = 0.81, 95% CI 0.36, 1.27, *p* < .001) and each additional year of age predicted lower scores (B = − 1.37, 95% CI − 1.81, − 0.94, *p* < .001). In DCHS, early life adversity was not associated with internalizing scores (B = − 0.18, *p* = .42). However, both age (B = − 1.62, 95% CI − 2.68, − 0.56, *p* = .003) and female sex (B = − 0.35, 95% CI − 0.70, − 0.01, *p* = .041) were associated with lower scores.

In BHRC, early life adversity was associated with higher externalizing scores (B = 3.48, 95% CI 2.05, 4.91, *p* < .001). Girls scored lower than boys (B = − 1.15, 95% CI − 1.72, − 0.58, *p* < .001). In SPRING, early life adversity was significantly associated with externalizing scores (B = 0.73, 95% CI 0.19, 1.26, *p* = .007) and each additional year of age predicted lower scores (B = − 1.86, 95% CI − 2.36, − 1.35, *p* < .001). In DCHS, early life adversity was not associated with externalizing scores (B = 0.3, *p* = .29). However, both age (B = − 3.87, 95% CI − 5.19, − 2.56, *p* < .001) and female sex (B = − 0.88, 95% CI − 1.3, − 0.45, *p* < .001) were associated with lower scores.

Sensitivity analyses using within-cohort standardized cumulative adversity scores yielded findings consistent with the primary analyses (see Supplementary Materials). Greater adversity was associated with greater behavioral difficulties across BHRC and SPRING cohorts. The DCHS cohort exhibited associations for externalizing behavior and total SDQ score. Standardized cumulative adversity scores were not associated with internalizing behavior in the DCHS cohort. These findings generally supported the primary analyses, with the overall pattern of stronger associations in BHRC and attenuated associations in SPRING and DCHS remaining largely consistent. However, some differences in the significance of specific outcomes across cohorts suggest that the observed associations may be sensitive to how adversity is operationalized.

### Secondary analyses

#### Early life adversity and age interactions

Linear regressions were conducted to examine the association between child age (centered), child sex, cohort, and the interaction of early life adversity and child age (centered), in predicting child behavior in each cohort.

**Fig. 2:**
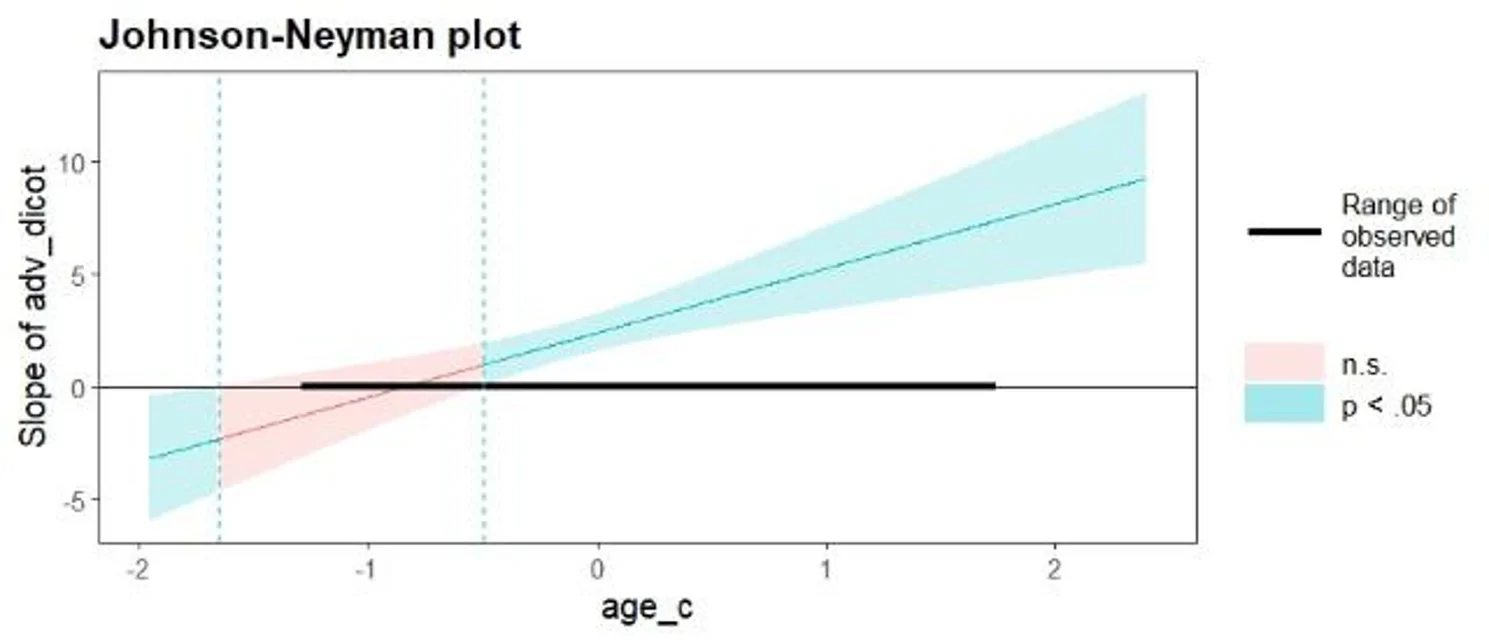
Early life adversity × child age interaction for SDQ total scores, showing that the association is not significant at younger ages and becomes significant beyond the threshold

The overall model for SDQ total score and ELA showed an association (*F*(6, 2413) = 232.2, *p* < .001). There was a main effect of early life adversity (B = 2.39, 95% CI 1.56, 3.23, *p* < .001) and child age (B = – 3.01, 95% CI – 4.44, – 1.57, *p* < .001). Girls scored lower than boys on SDQ total score (B = – 0.95, 95% CI – 1.44, – 0.47, *p* < .001). SPRING and DCHS participants had lower SDQ total scores (SPRING: B = – 10.81, 95% CI – 11.47, – 10.15, *p* < .001; DCHS: B = – 6.13, 95% CI – 6.76, – 5.5, *p* < .001). There was a strong association of early life adversity × child age interaction (B = 2.87, 95% CI 1.4, 4.34, *p* < .001), which can be seen in Fig. 2. Johnson–Neyman analysis indicated that the association became between adversity and internalizing behavior was significant at higher ages (centered > − 0.50), corresponding to approximately 8.16 years. No significant association was observed at younger ages within the sample.

**Fig. 3:**
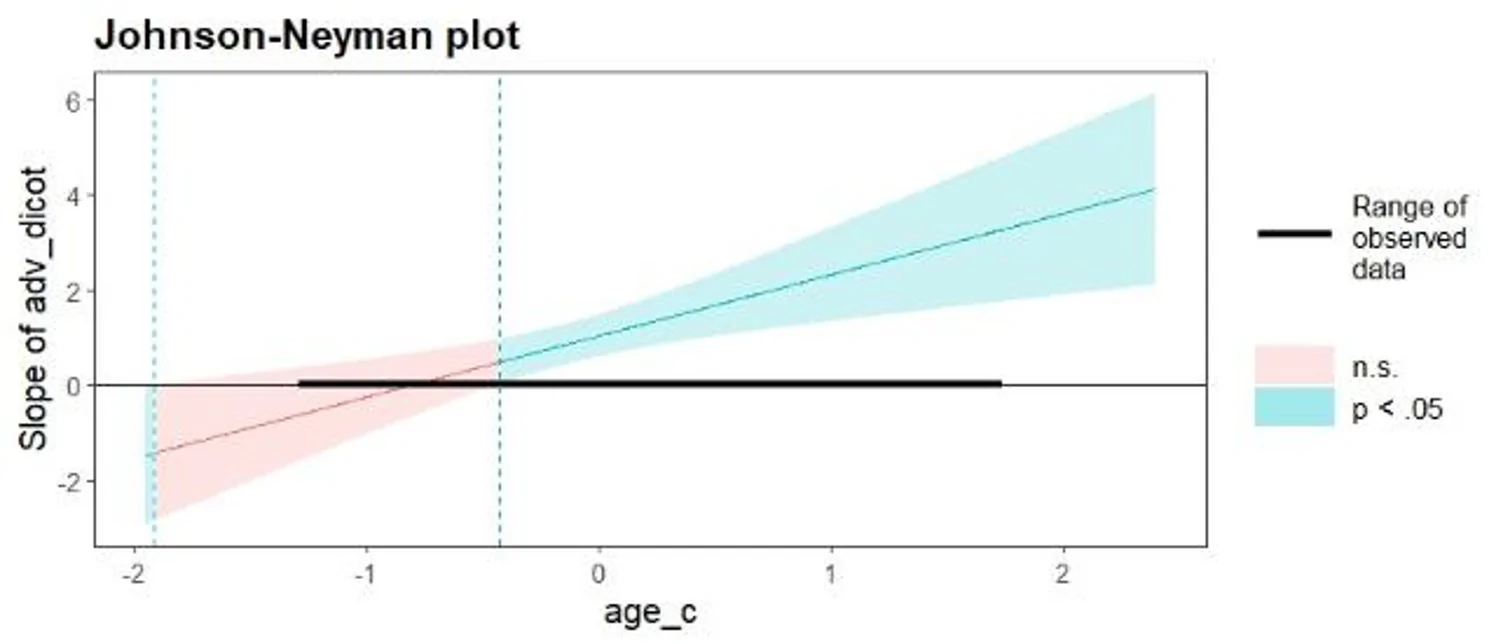
Early life adversity × child age interaction for internalizing scores showing that the association is not significant at younger ages and becomes significant beyond the threshold

The overall model for internalizing behavior and ELA showed an association (*F*(6, 2413) = 208.9, *p* < .001). There was a main effect of early life adversity (B = 1.03, 95% CI 0.59, 1.47, *p* < .001) and child age (B = – 1.21, 95% CI – 1.96, – 0.45, *p* = .002). SPRING and DCHS participants had lower internalizing behavior scores (SPRING: B = – 4.98, 95% CI – 5.33, – 4.63, *p* < .001; DCHS: B = – 3.83, 95% CI – 4.16, – 3.50, *p* < .001). There was an association of early life adversity × child age interaction (B = 1.29, 95% CI 0.51, 2.06, *p* = .001), which can be seen in Fig. 3. Johnson–Neyman analysis indicated that the association became between adversity and internalizing behavior was significant at higher ages (centered > − 0.43), corresponding to approximately 8.21 years. No significant association was observed at younger ages within the sample.

**Fig. 4:**
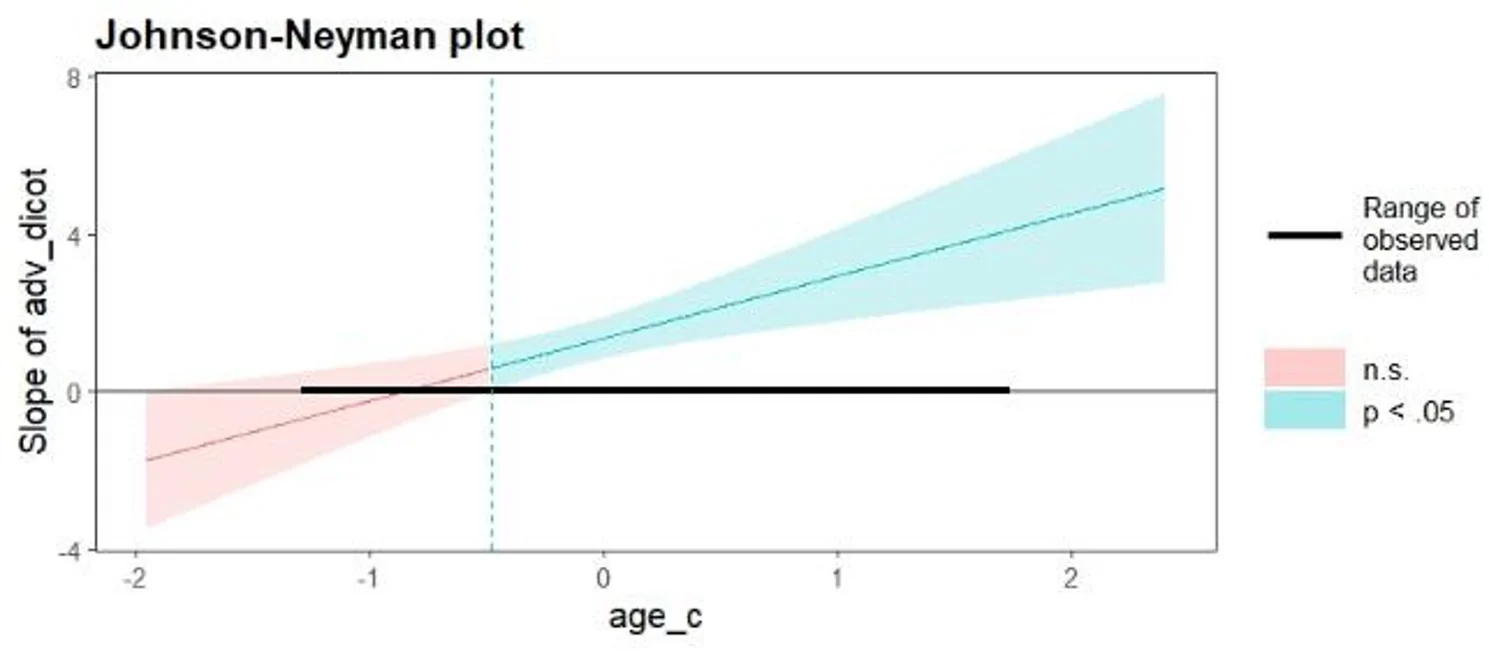
Early life adversity × child age interaction for externalizing scores showing that the association is not significant at younger ages and becomes significant beyond the threshold

The overall model for externalizing behavior and ELA showed an association (*F*(6, 2413) = 160.5, *p* < .001). There was a main effect of early life adversity (B = 1.36, 95% CI 0.84, 1.89, *p* < .001) and child age (B = – 1.8, 95% CI – 2.7, – 0.89, *p* = .006). Girls scored lower than boys on externalizing behaviors (B = – 0.85, 95% CI – 1.15, – 0.55, *p* < .001). SPRING and DCHS participants had lower externalizing behavior scores (SPRING: B = – 5.83, 95% CI – 6.24, – 5.41, *p* < .001; DCHS: B = – 2.30, 95% CI – 2.7, – 1.91, *p* < .001). There was a strong association of early life adversity × child age interaction (B = 1.59, 95% CI 0.66, 2.51, *p* < .001), which can be seen in Fig. 4. Johnson–Neyman analysis indicated that the association between adversity and externalizing behavior was significant only at higher ages (centered > − 0.48), corresponding to approximately 8.18 years. No significant association was observed at younger ages within the sample.

#### Early life adversity and cohort interactions

Linear regressions were conducted to examine the association between age, sex, cohort, and the interaction between early life adversity and cohort, on child behavior.

**Fig. 5:**
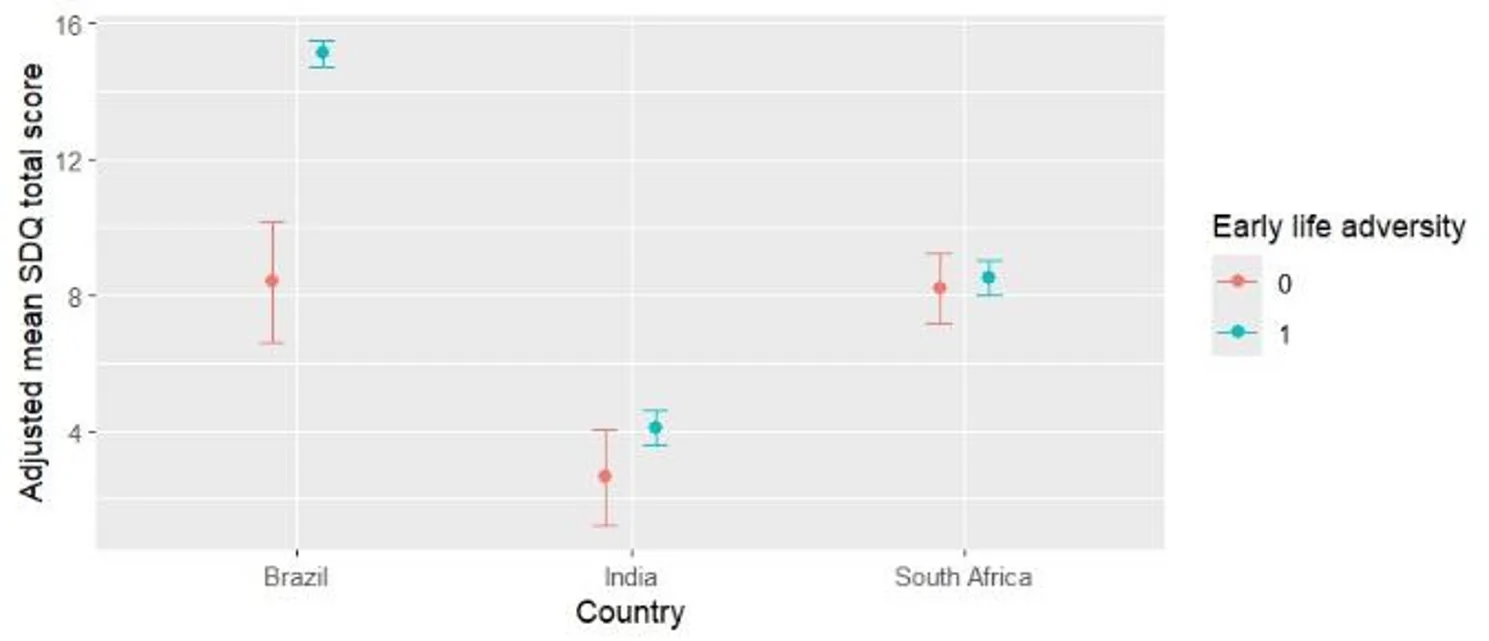
Estimated marginal means of SDQ total scores from a linear model including early life adversity, country, their interaction, and child age and sex. Points show model-adjusted means; error bars represent 95% confidence intervals

There was an association for the overall model for SDQ total scores (*F*(7, 2412) = 203.7, *p* < .001). There was a main effect of early life adversity (B = 6.76, 95% CI 4.97, 8.54, *p* < .001) and child sex, with girls showing lower SDQ total scores than boys (B = – 0.99, 95% CI – 1.47, – 0.51, *p* < .001). Compared to BHRC, SPRING had lower scores (B = − 5.75, 95% CI – 8.01, – 3.49, *p* < .001), while DCHS did not differ (B = − 0.2, *p* = .85). There was an early life adversity × country interaction, and the effect of early life adversity was reduced in SPRING and DCHS participants, relative to BHRC participants (SPRING: B = – 5.29, 95% CI – 7.62, – 2.97, *p* < .001; DCHS: B = – 6.43, 95% CI – 8.54, – 4.31, *p* < .001). Adjusted marginal means of SDQ total scores were higher in the adversity group than the no– adversity group across countries, with variation in effect size highlighting an early life adversity × country interaction (see Fig. 5).

**Fig. 6:**
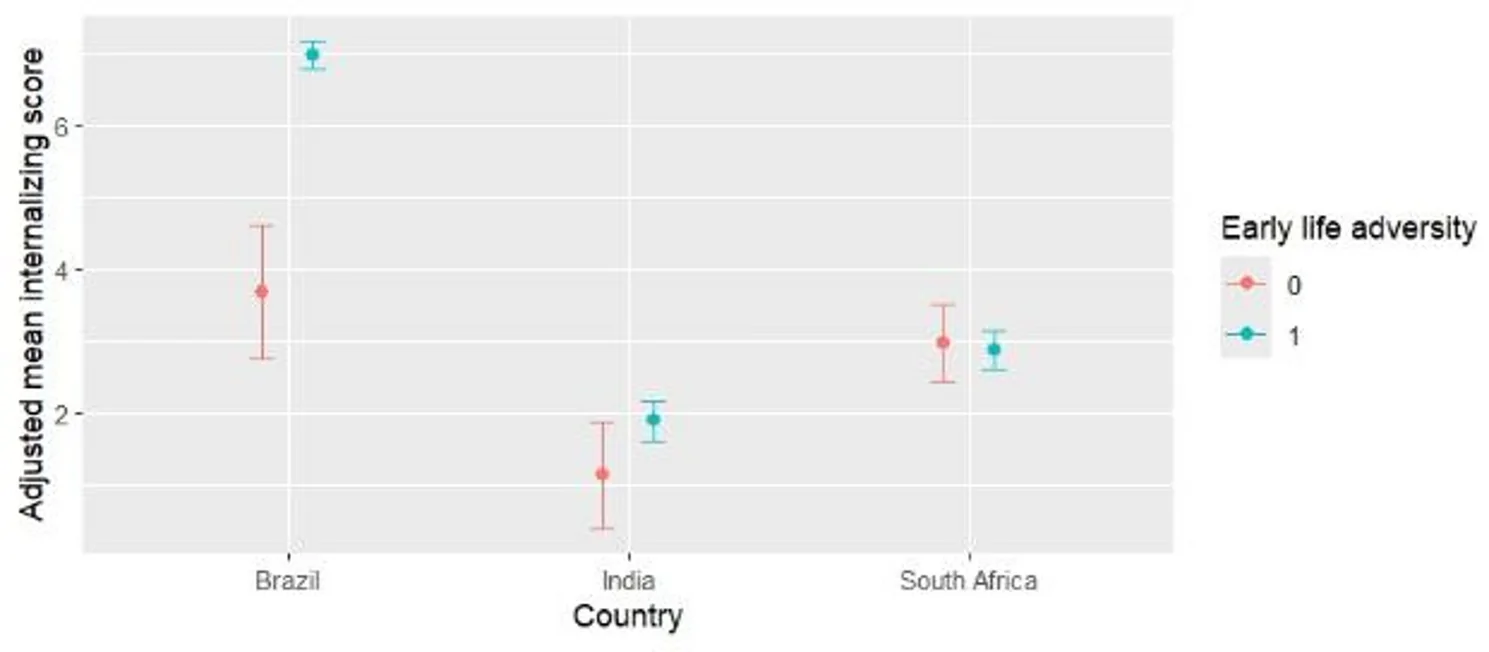
Estimated marginal means of internalizing scores from a linear model including early life adversity, country, their interaction, and child age and sex. Points show model-adjusted means; error bars represent 95% confidence intervals

There was an association for the overall model for internalizing behaviors (*F*(7, 2412) = 184.5, *p* < .001). There was a main effect of early life adversity (B = 3.31, 95% CI 2.37, 4.25, *p* < .001). Compared to BHRC, SPRING exhibited lower internalizing scores (B = – 2.53, 95% CI – 3.72, – 1.34, *p* < .001), while DCHS did not differ (B = – 0.7, *p* = .21). There was an early life adversity × country interaction, and the effect of early life adversity was reduced in SPRING and DCHS participants, relative to BHRC participants (SPRING: B = – 2.55, 95% CI – 3.77, – 1.33, *p* < .001; DCHS: B = – 3.41, 95% CI – 4.53, – 2.30, *p* < .001). Adjusted marginal means of internalizing scores were higher in the adversity group than the no-adversity group across countries, with variation in effect size highlighting an early life adversity × country interaction (see Fig. 6).

**Fig. 7:**
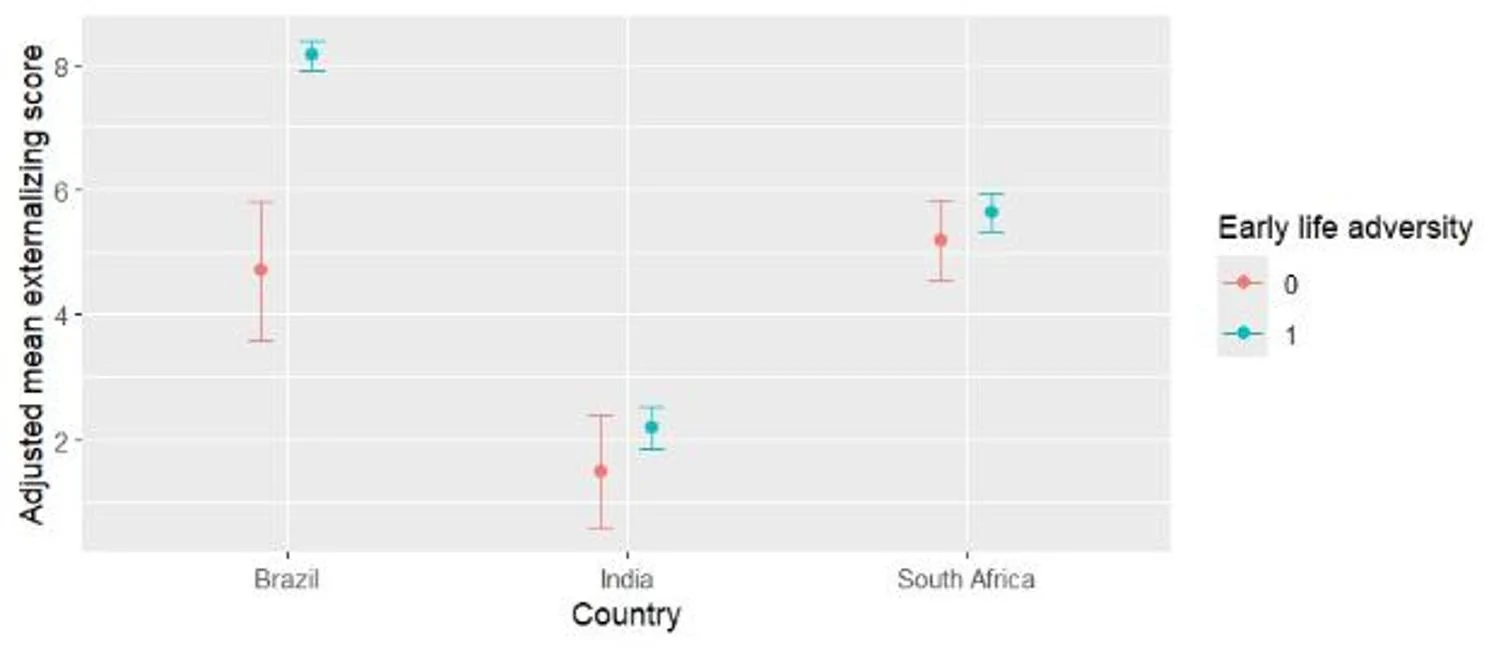
Estimated marginal means of externalizing scores from a linear model including early life adversity, country, their interaction, and child age and sex. Points show model-adjusted means; error bars represent 95% confidence intervals

There was an association for the overall model for externalizing behaviors (*F*(7, 2412) = 139.4, *p* < .001). There was a main effect of early life adversity (B = 3.45, 95% CI 2.33, 4.58, *p* < .001) and child age (B = – 0.31, 95% CI – 0.56, – 0.06, *p* = .02). There was a main effect for child sex, with girls showing fewer externalizing behaviors than boys (B = – 0.87, 95% CI – 1.17, – 0.56, *p* < .001). Compared to BHRC, SPRING exhibited lower externalizing scores (B = – 3.22, 95% CI – 4.64, – 1.79, *p* < .001), while DCHS did not differ (B = 0.5, *p* = .46). There was an early life adversity × country interaction, and the effect of early life adversity was reduced in SPRING and DCHS participants, relative to BHRC participants (SPRING: B = – 2.74, 95% CI – 4.21, – 1.28, *p* < .001; DCHS: B = – 3.01, 95% CI – 4.35, – 1.68, *p* < .001). Adjusted marginal means of externalizing scores were higher in the adversity group than the no-adversity group across countries, with variation in effect size highlighting an early life adversity × country interaction (see Fig. 7).

#### Early life adversity and child sex interactions

Linear regressions were conducted to examine the association between child age, cohort, and the interaction between early life adversity and child sex, on child behavior.

There was an association for the overall model for total SDQ score (*F*(6, 2413) = 228.9, *p* < .001). There was a main effect of early life adversity (B = 2.49, 95% CI 1.40, 3.58, *p* < .001). Compared to BHRC, SPRING exhibited lower SDQ total scores (B = – 10.78, 95% CI – 11.45, – 10.12, *p* < .001), as well as DCHS (B = – 6.15, 95% CI – 6.78, – 5.52, *p* < .001). There was no evidence that the association between early life adversity and SDQ total scores differed by sex (B = – 1.15, 95% CI – 2.74, 0.45, *p* = .16).

There was an association for the overall model for internalizing behavior (*F*(6, 2413) = 206.4, *p* < .001). There was a main effect of early life adversity (B = 1.01, 95% CI 0.44, 1.58, *p* < .001). Compared to BHRC, SPRING exhibited lower internalizing scores (B = – 4.97, 95% CI – 5.31, – 4.62, *p* < .001), as well as DCHS (B = – 3.84, 95% CI – 4.17, – 3.50, *p* < .001). There was no evidence that the association between early life adversity and internalizing scores differed by sex (B = – 0.38, 95% CI – 1.22, 0.46, *p* = .46).

There was an association for the overall model for externalizing behavior (*F*(6, 2413) = 158.3, *p* < .001). There was a main effect of early life adversity (B = 1.48, 95% CI 0.80, 2.16, *p* < .001) and age (B = – 0.31, 95% CI – 0.56, – 0.06, *p* = .017). Compared to BHRC, SPRING exhibited lower externalizing scores (B = – 5.81, 95% CI – 6.523, – 5.40, *p* < .001), as well as DCHS (B = – 2.32, 95% CI – 2.71, – 1.92, *p* < .001). There was no evidence that the association between early life adversity and externalizing scores differed by sex (B = – 0.77, 95% CI – 1.77, 0.23, *p* = .13).

## Discussion

In cohort-specific analyses, cumulative early life adversity in the first two years of life was associated with higher SDQ total, internalizing, and externalizing scores at age 7–10. The strength of these associations varied by cohort (strongest in BHRC, attenuated or non-significant in SPRING and DCHS, respectively), indicating context-dependent effects. Additionally, ELA × age interactions suggested larger effects at younger ages, and sex differences were observed in some cohorts.

Our finding that early life adversity was associated with higher behavioral scores for the SDQ total score, internalizing, and externalizing behaviors is consistent with prior work in high-income countries [42, 43]. This includes research on translational models [4], clinical cohorts, and community cohorts [44–46]. Large, high-income cohorts have reported that multiple childhood adversities were associated with greater internalizing and externalizing behaviors in mid-childhood [42, 43]. Thus, the association between early life adversity and psychopathology is already evident in mid-childhood. As the standardized effect sizes for these associations are relatively small in this study, the findings may not be clinically relevant on an individual level. However, given the high prevalence of adversity exposures in the cohorts, small shifts in behavioral risk may translate into meaningful population-level burden [47].

Cohort-specific findings were observed. These differences indicate that the association between early life adversity and child behavior is not uniform across low- and middle-income settings. Our findings indicated that BHRC participants exhibited a greater association of ELA with behavior outcomes relative to the SPRING cohort. In the BHRC, the stronger associations may partly reflect the sampling methods, as children with elevated psychiatric risk were oversampled. This design may have increased the prevalence of early life adversity exposure and/or behavioral problems within the sample. In the SPRING cohort, associations were attenuated but age effects were observed. As the SPRING cohort included a greater prevalence of extended family home systems [30], the shared caregiving by multiple family members may buffer the impact of adversity on child behavior. The DCHS cohort did not exhibit an association between ELA and behavior outcomes but instead highlighted that child age and sex were predictors of behavioral outcomes. These findings may reflect differences in recruitment methods or adversities captured. These cohort-specific differences may reflect a combination of contextual risk factors [48], a variation in early life adversity exposure, or differences in cultural expression and reporting of child behavior. Interpretation of between-cohort differences is constrained by differences in measurement and incomplete equivalence of behavioral assessments.

We found that the association of early life adversity with behavior is stronger at older ages. This is consistent with a prior literature that discusses the development of resilience over time [49, 50]. We found that sex differences differed by country. In the BHRC, girls scored lower than boys on SDQ total score and externalizing behavior, in DCHS girls scored lower than boys on SDQ total score, internalizing, and externalizing behaviors, and in SPRING there were differences in SDQ scores by sex. However, we found no evidence that the association between early life adversity and behavioral outcomes differed by sex in the full cohort. While sex differences in behavioral outcomes have been observed in the literature, these effects are often small [51].

Several mechanisms may explain the above findings. Early life adversity may influence neurodevelopment through stress-related alterations in hypothalamic–pituitary–adrenal axis functioning and inflammatory pathways [52]. Smith and Pollak [53] highlight that exposure to early life adversity is associated with persistent effects on the prefrontal-hippocampal-amygdala circuit. As this network underpins emotion processing and self-regulation, such alterations may increase vulnerability to problem behavior [54]. Early life adversity also influences brain development through neuroplasticity in early life [55, 56]. Psychosocial pathways are also likely important, including disrupted caregiving, reduced cognitive stimulation, and chronic environmental stress, which may shape behavioral development through direct and indirect effects of caregiving. For example, adversity may compromise caregiver sensitivity and responsiveness to the child, which has been linked to increased internalizing and externalizing behaviors in children [57].

The cohort-specific findings highlight that interventions addressing early life adversity and child behavioral outcomes should be context-specific rather than uniform across settings. Interventions may target the reduction of early life adversity or the mitigation of its downstream effects on child development. Interventions may include early maternal mental health screening and treatment, parenting interventions to enhance caregiver sensitivity and responsiveness, poverty alleviation strategies (e.g., cash transfers or food security programs), and community-based early childhood development services that promote cognitive and socioemotional stimulation. A number of low-cost and scalable programs indicate that successful interventions can be implemented in low-resource settings [58–61]. Intervention feasibility is likely to differ across contexts depending on health system capacity, resource availability, and the predominant types of adversity. For example, in settings where socioeconomic deprivation is the primary adversity, structural interventions targeting poverty and food insecurity may be most impactful, whereas in contexts with elevated interpersonal or psychosocial risk, parenting and mental health interventions may be more appropriate. The heterogeneity observed across cohorts in the present study suggests that intervention strategies should prioritize locally salient risk factors and leverage existing service platforms, rather than adopting a uniform approach across countries.

Some limitations should be emphasized. First, the adversity variable was heterogeneous between cohorts, therefore, to mitigate heterogeneity in the adversity variable, the adversity variable was dichotomized. Dichotomization may have reduced variability, however, sensitivity analyses using continuous cumulative adversity z-scores yielded results consistent with the primary analyses. Missing adversity items were coded as absence of exposure to maximize sample retention and maintain comparability across cohorts. However, if missingness was associated with adversity exposure, this approach may have underestimated cumulative adversity and attenuated the observed associations. Additionally, the adversity measure does not distinguish between transient and chronic exposures and may therefore partly capture ongoing adversity beyond the early developmental window. Second, this study examined adversity experienced in the first two years of life and adversity experienced after 2 years of age was not included. Therefore, it is not possible to account for the cumulative, time-varying, or potentially interacting effects of adversities experienced beyond infancy, which may also be critical for behavioral outcomes. Third, measurement invariance did not hold across cohorts, indicating that results may not be directly comparable, therefore cohort differences should be interpreted cautiously. Finally, the BHRC employed an oversampling strategy targeting children at psychiatric risk which likely increased the prevalence of adversity exposure and behavioral problems within the sample. This sampling design may have inflated the observed adversity–behavior associations relative to SPRING and DCHS cohorts. These findings should be interpreted with caution.

In conclusion, in cohorts from the Global South, while there is an association between early life adversity and psychopathology, there are also important context-dependent differences across cohorts. Early life trauma may be particularly important to identify, and culturally tailored interventions may be needed. Future work should prioritize the development of a minimal, harmonized set of adversity indicators that can be implemented across diverse LMIC cohorts while retaining sensitivity to context-specific exposures. Longitudinal designs incorporating repeated and harmonized assessments will be critical for disentangling developmental timing effects from cohort-specific measurement differences.

## Supplementary Information

Below is the link to the electronic supplementary material.

Supplementary Material 1.

Supplementary Material 2.

## Data Availability

The datasets used and/or analyzed during the current study are available from the corresponding author on reasonable request.
