# Supplementary material for: Association of early life adversity with internalizing and externalizing behavior across 3 low- and middle-income countries
Source: Child Adolesc Psychiatry Ment Health. 2026 Jul 24;20:148. doi: 10.1186/s13034-026-01139-9 (PMC13715187; doi:10.1186/s13034-026-01139-9)
Supplement: Supplementary file 1 [file 13034_2026_1139_MOESM2_ESM.docx]

**Supplementary Materials**

**Association of early life adversity with internalizing and externalizing behavior across 3 low- and middle-income countries**

Sheri-Michelle Koopowitz^1^, Richard Shadwell^3,4^, Nadia Hoffman^1^, Heather J Zar^3,4^, Giovanni Salum^5,6^, Pedro M Pan^7^, Gauri Divan^8^, Supriya Bhavnani^8^, Dan J Stein^1, 2^

Affiliations

1. Neuroscience Institute, Faculty of Health Sciences, University of Cape Town
2. South African Medical Research Council (SAMRC) Unit on Risk and Resilience in Mental Disorders, Cape Town, South Africa
3. Department of Paediatrics & Child Health, Red Cross War Memorial Children’s Hospital, University of Cape Town, South Africa
4. South African Medical Research Council (SAMRC) Unit on Child and Adolescent Health, Cape Town, South Africa
5. Global Programs, Child Mind Institute, New York, USA
6. Universidade Federal do Rio Grande do Sul, Hospital de Clínicas, Porto Alegre, Brazil
7. Universidade Federal de São Paulo (UNIFESP)
8. Child Development Group, Sangath, India

**Results**

**Exploratory findings**

Due to measurement invariance not holding, models examining early life adversity exposure and child behavior were exploratory in nature. Table 3 highlights the standardized estimates (β) for the models exploring early life adversity and behavior. Exposure to early life adversity was associated with higher total SDQ scores (B = 1.97, 95% CI: 1.16, 2.78, *p* < .001). Girls had lower total SDQ scores than boys (B = –0.95, 95% CI: –1.43, –0.47, *p* < .001). Children from India (B = –10.79, 95% CI: –11.45, –10.13, *p* < .001) and South Africa (B = –6.14, 95% CI: –6.77, –5.51, *p* < .001) had lower scores than children from Brazil. The final model explained 36% of the variance in SDQ scores (Adjusted *R²* = .361, *F*(5, 2414) = 274.1, *p* < .001).

Exposure to early life adversity was associated with higher internalizing scores (B = 0.84, 95% CI: 0.41, 1.27, *p* < .001). Children from India (B = –4.97, 95% CI: –5.32, –4.62, *p* < .001) and South Africa (B = –3.83, 95% CI: –4.16, –3.5, *p* < .001) had lower internalizing scores than children from Brazil. The adjusted model explained 34% of the variance in internalizing scores (Adjusted *R²* = .338, *F*(5, 2414) = 247.6, *p* < .001).

Exposure to early life adversity was associated with higher externalizing scores (B = 1.13, 95% CI: 0.62, 1.64, *p* < .001). Each additional year of age was associated with lower externalizing scores (B = –0.31, 95% CI: –0.56, –0.06, *p* = .017), and girls had lower scores than boys (B = –0.85, 95% CI: –1.15, –0.55, *p* < .001). Compared with children from Brazil, children from India (B = –5.82, 95% CI: –6.23, –5.40, *p* < .001) and South Africa (B = –2.31, 95% CI: –2.7, –1.19, *p* < .001) had lower externalizing scores. The adjusted model explained 28% of the variance in externalizing scores (Adjusted *R²* = .280, *F*(5, 2414) = 189.5, *p* < .001).

| Table 3. *Standardized regression coefficients for associations between adversity and behavior outcomes* | | | | | | |
| --- | --- | --- | --- | --- | --- | --- |
|  | SDQ total score | | Internalizing | | Externalizing | |
|  | β [95% CI] | p value | β [95% CI] | p value | β [95% CI] | p value |
| Adversity | 0.08 [0.05, 0.11] | **< .001** | 0.07 [0.03, 0.10] | **< .001** | 0.08 [0.04, 0.11] | **< .001** |
| Age (years) | -0.03 [-0.06, 0.01] | .129 | -0.00 [-0.04, 0.04] | .979 | -0.05 [-0.09, -0.01] | **.017** |
| Sex (Girl) | -0.13 [-0.19, -0.06] | **< .001** | -0.03 [-0.09, 0.04] | .437 | -0.19 [-0.26, -0.12] | **< .001** |
| Country (India) | -1.43 [-1.52, -1.35] | **< .001** | -1.28 [-1.37, -1.19] | **<.001** | -1.31 [-1.40, -1.21] | **< .001** |
| Country (South Africa) | -0.82 [-0.90, -0.73] | **< .001** | -0.98 [-1.07, -0.90] | **<.001** | -0.52 [-0.61, -0.43] | **< .001** |

*Note.* Coefficients are standardized regression estimates (β).

**Analysis using within-cohort cumulative adversity z-scores**

| Outcome | BHRC | | SPRING | | DCHS | |
| --- | --- | --- | --- | --- | --- | --- |
| Externalizing | β = 1.32 | p < .001 | β = 0.47 | p < .001 | β = 0.29 | p = .006 |
| Internalizing | β = 1.39 | p < .001 | β = 0.36 | p < .001 | β = 0.16 | p = .061 |
| SDQ total score | β = 2.71 | p < .001 | β = 0.83 | p < .001 | β = 0.46 | p = .005 |

**Missing data analysis (DCHS)**

| Variable | RiskRatio | p | LCL | UCL | sig | mean_in_sample | mean_out_sample | N_in_sample | N_out_sample |
| --- | --- | --- | --- | --- | --- | --- | --- | --- | --- |
| Maternal_HIV positive | 1,221149 | 3,45E-05 | 1,110973 | 1,342251 | *** | 0,252825 | 0,158621 | 708 | 435 |
| Child sex - Male | 0,939832 | 0,180606 | 0,85822 | 1,029205 |  | 0,497175 | 0,537931 | 708 | 435 |
| Household_incomeR1000-5000/m | 0,988659 | 0,819091 | 0,896597 | 1,090174 |  | 0,528249 | 0,511521 | 708 | 434 |
| Household_income>R5000/m | 0,876378 | 0,101077 | 0,74849 | 1,026116 |  | 0,125706 | 0,163594 | 708 | 434 |
| MaternalEducation (At least secondary or higher) | 0,857319 | 0,002036 | 0,777436 | 0,94541 | ** | 0,355932 | 0,450575 | 708 | 435 |
| parent_working | 0,948998 | 0,258921 | 0,866553 | 1,039287 |  | 0,49435 | 0,528736 | 708 | 435 |
| Asset sum | 0,97034 | 0,009598 | 0,948481 | 0,992703 | ** | 6,717514 | 7,03908 | 708 | 435 |
| Married/cohabiting | 0,964316 | 0,446166 | 0,878253 | 1,058813 |  | 0,39548 | 0,418391 | 708 | 435 |
| Preterm <37wks | 0,839884 | 0,015622 | 0,729093 | 0,967511 | * | 0,142655 | 0,202299 | 708 | 435 |
| Prenatal_alcohol_CompositeExposure | 1,038214 | 0,567173 | 0,913062 | 1,18052 |  | 0,132653 | 0,120735 | 686 | 381 |
| Prenatal_smoking_Composite | 0,936751 | 0,221227 | 0,843643 | 1,040135 |  | 0,269774 | 0,304147 | 708 | 434 |
| Maternal_age_birth | 1,010045 | 0,010753 | 1,002317 | 1,017832 | * | 27,24294 | 26,37241 | 708 | 435 |
| EPDS_threshold_Above threshold | 0,970107 | 0,598853 | 0,866384 | 1,086246 |  | 0,232964 | 0,247934 | 631 | 363 |
| BDI_total_Above threshold | 0,942721 | 0,362606 | 0,830296 | 1,070368 |  | 0,17965 | 0,203857 | 629 | 363 |
| Childhood_adversity | 0,973994 | 0,022038 | 0,952272 | 0,996213 | * | 1,927966 | 2,291667 | 708 | 168 |
| SDQ_total | 1,003852 | 0,108025 | 0,999156 | 1,008571 |  | 8,516949 | 7,827586 | 708 | 87 |
